# Fungal spores in Caribbean mangrove sediments, dataset from southeastern Mexico

**DOI:** 10.1016/j.dib.2023.109631

**Published:** 2023-09-28

**Authors:** Cynthia Karina Can-Canales, Gerald A. Islebe, Alicia Carrillo-Bastos, Nuria Torrescano-Valle, Alejandro Antonio Aragón-Moreno

**Affiliations:** a El Colegio de la Frontera Sur, Unidad Chetumal, Chetumal, Mexico; b Tecnológico Nacional de México / IT de Chetumal, Chetumal, Mexico

**Keywords:** Palynology, Fungal spores, Microfossil, Paleoecology, Mangroves, Paleontology

## Abstract

Most paleoecological investigations use different biotic or abiotic proxies for climate and environmental reconstructions. Although fossil pollen is one of the most used biological proxies, Non-Pollen Palynomorphs (NPPs), especially fungal spores and tissues, have an underestimated potential to infer local and regional climate dynamics. This dataset describes the most common Non-pollen palynomorphs of fungal origin from mangrove sediments in the Caribbean Sea, southeastern Mexico. A detailed descriptive Atlas is presented, with light micrographs taken from routine pollen slides in paleoecological reconstructions. Microphotographs were included to facilitate their identification. A total of 59 spores, 4 tissues, 2 hyphae, and 11 unidentified fungal palynomorphs are described.

Specifications Table

**Table:** 

Subject	Earth and Planetary Sciences - Paleontology
Specific subject area	Non-pollen Palynomorphs - Fungal spores and tissues
Type of data	Image and Table
How the data were acquired	Micrographs were acquired via Carl Zeiss - Primo Star light microscope with 5 Megapixels integrated camera. Images were obtained via 400X magnification and processed with AxioVision software. Fungal NPP counts and identification was made from prepared pollen slides at 2.5cm intervals of a 150cm long sediment core retrieved at Laguna Cementerio, Mexico.
Data format	Raw
Description of data collection	Fungal spores, tissues, and hyphae were identified based on morphometric measurements and analysis of apertures, wall thickness, shape, number of segments, and texture. Various NPPs identification keys were used [1], [2], [3], [4], [5], [6], [7]. The taxa were named with the abbreviation ECO (El Colegio de la Frontera Sur) followed by a numbering.
Data source location	*·* City/Town/Region: Laguna Cementerio, Caribbean Sea, southeastern Mexico (Fig. 1) · Country: Mexico · Latitude and longitude (and GPS coordinates, if possible) for collected samples/data: 18°15’28.6” N; 87°50’37.1” W
Data accessibility	Repository name: Mendeley Data Data identification number: DOI:10.17632/9gmhxx5t2g.2 Direct URL to data: https://data.mendeley.com/datasets/9gmhxx5t2g/4

## 1 Value of the Data

• Fungal spore and tissue atlas assist in the identification of fungal-origin NPPs in mangrove systems used for paleoenvironmental research
• Paleoecologists could use this dataset to reinforce paleoclimate and paleoecological interpretations
• Fungal NPPs are underestimated climate proxies; a correct Fungal NPPs identification could reinforce paleoclimate and paleoecological reconstructions in the Caribbean
• Fungal spore and tissue atlas are virtually nonexistent for Caribbean mangroves

## 2 Objective

**Fig. 1:**
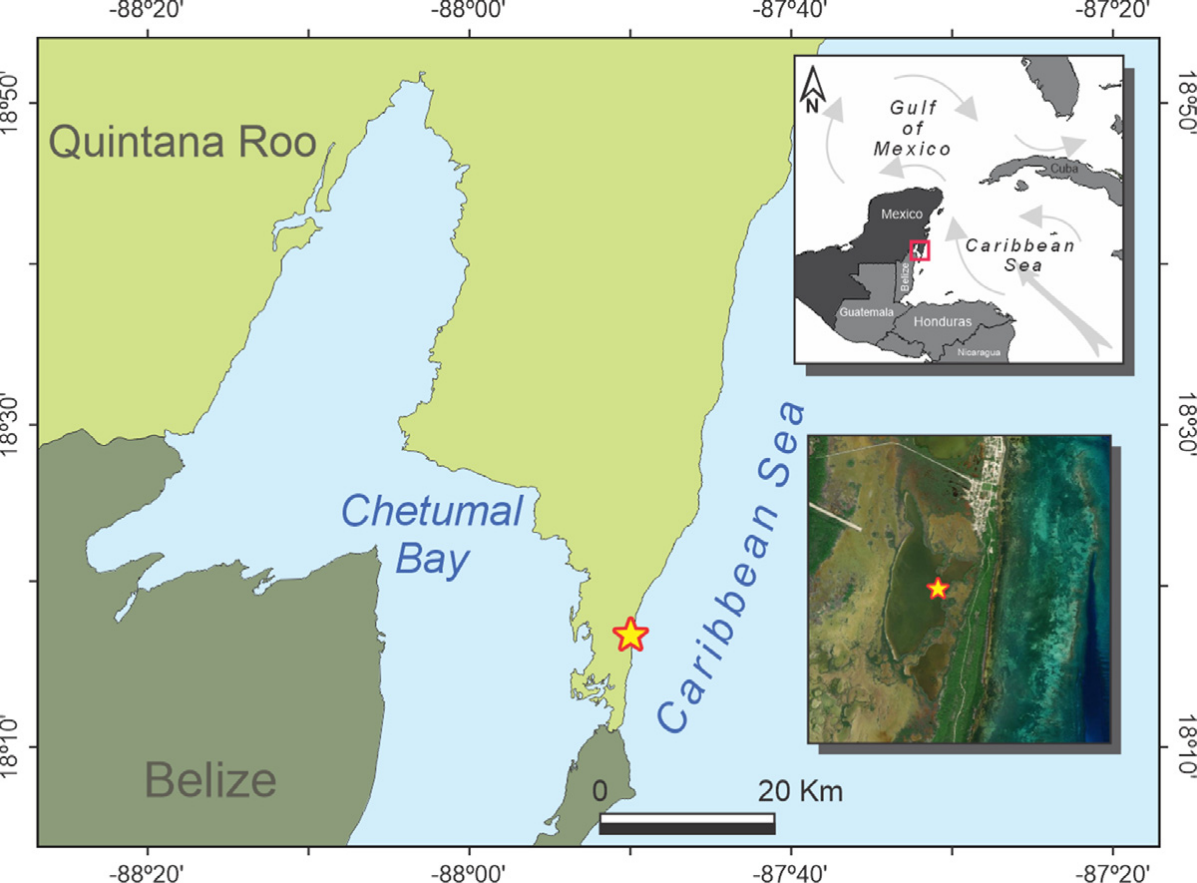
Laguna Cementerio location, southeastern Mexico.

Our main objective is to improve Late Holocene paleoenvironmental reconstructions of mangroves of the Caribbean basin. Proper fungal spores, tissue, or structure identification could reinforce, or complement, climate and ecological interpretations from other proxies in mangrove sediments. This dataset also aims to offer paleoecologists easy-access fungal origin non-pollen palynomorphs description with their environmental affinities (Fig. 1).

## 3 Data Description

Fungal spores and tissues have been used as complementary environmental indicators in paleoenvironmental research since the early 1970s [8]. They are some of the most common NPPs found in mangrove sediments that exhibit environmental conditions caused by erosion, marine intrusion, anthropogenic activity, humidity, or salinity [3,9]. Although several paleoenvironmental studies have reported fungal presence and concentrations as environmental indicators [10], [11], [12], identification keys in Mexico are notably rare [13], and virtually non-existent in the Yucatan peninsula, complicating their use as an effective proxy for specific environmental conditions. Fungal activity in mangroves is relevant for nutrient cycling and ecosystem regulation. Fungi in mangrove sediments participate in various ecological processes. Key roles of fungi in mangrove sediments include decomposition, symbiotic relationships, nutrient cycling and soil structure.

**Plate 1:**
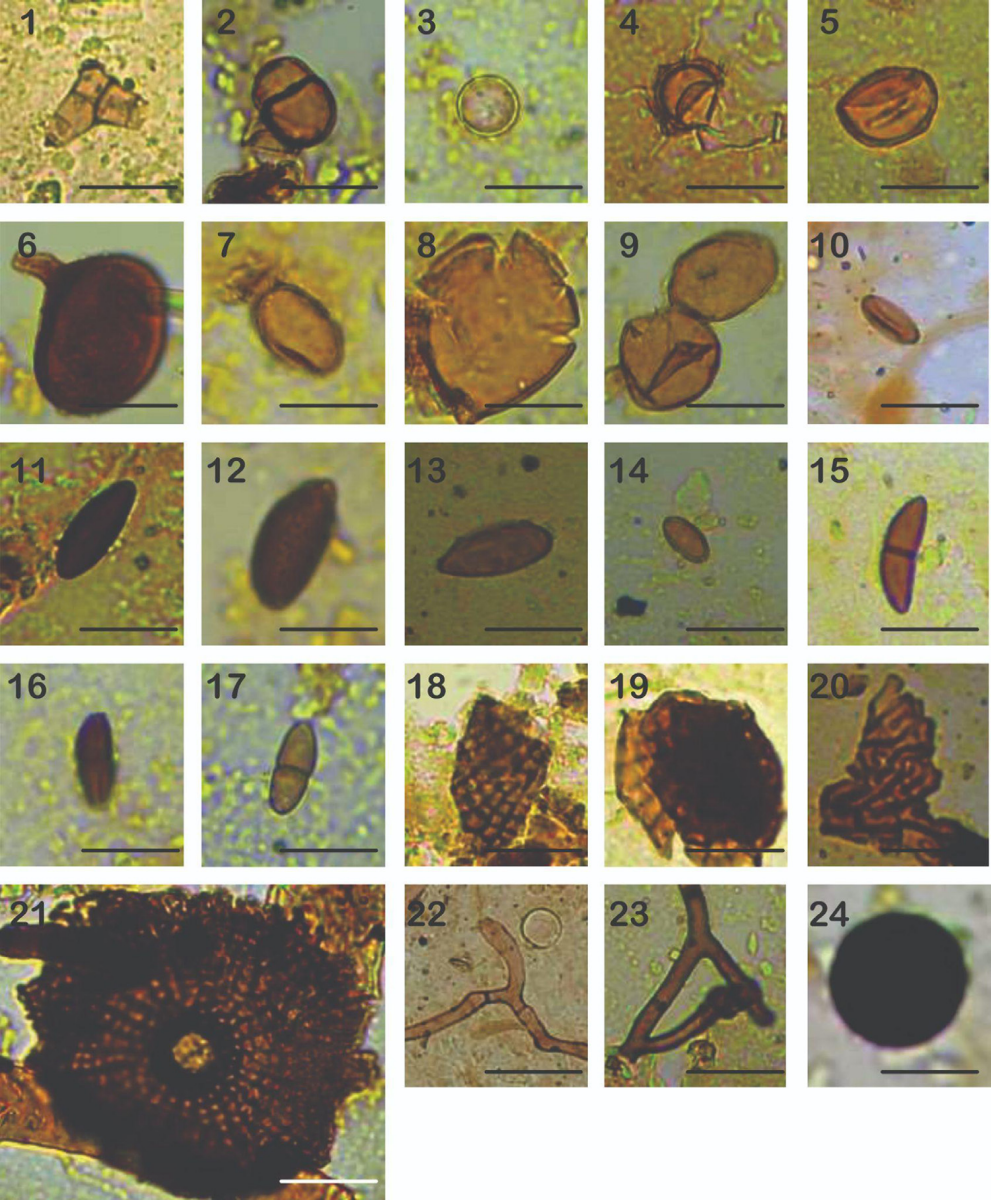
1: *Curvularia* sp.; 2-9: *Glomus* sp.; 10-14: Xylariaceae; 18-21: *Mycrothyrium* sp.; 22-23: Hyphae; 24: Ascomycota spore. Scale bar of 20 µm.

**Plate 2:**
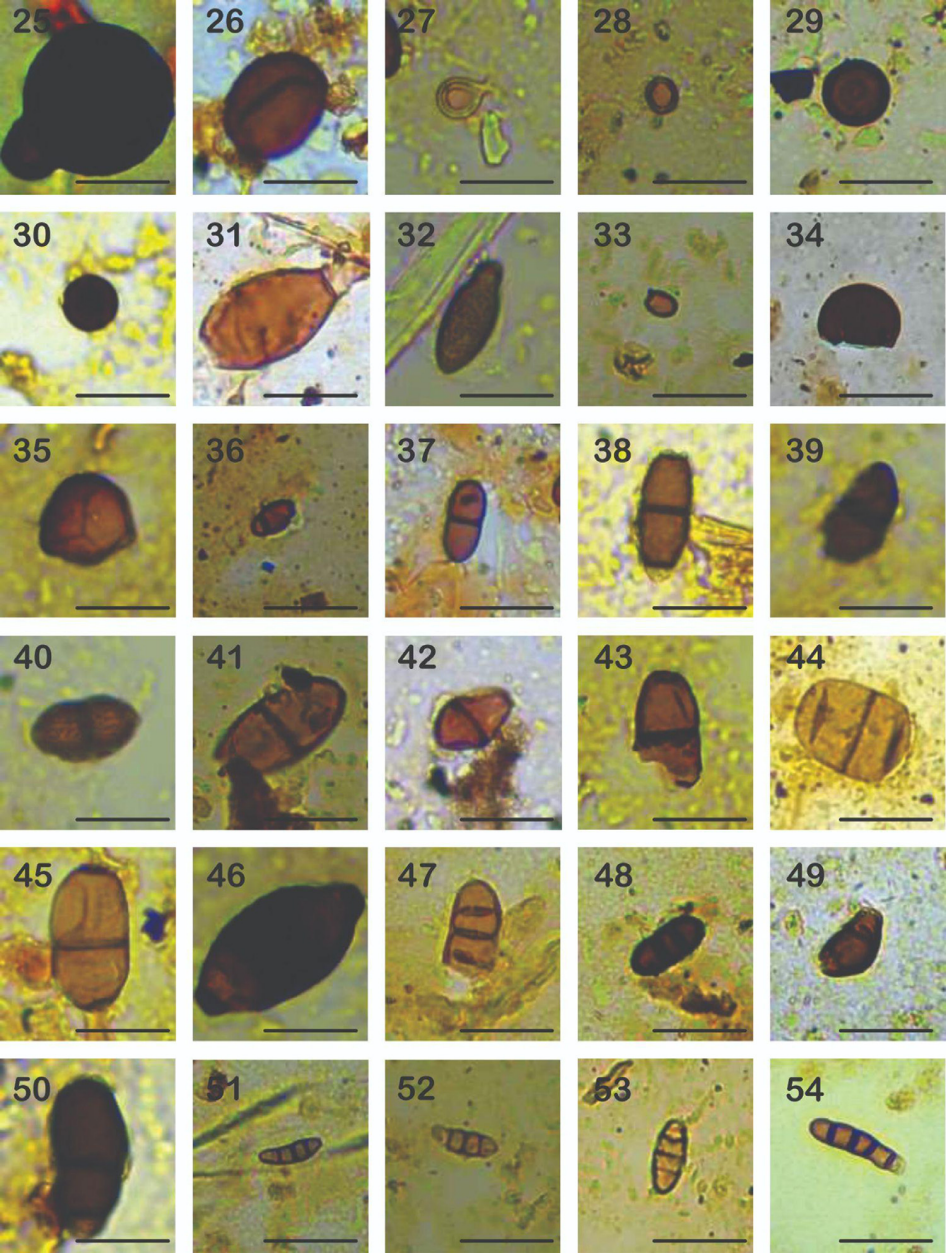
25-54: Spore of Ascomycota group. Scale bar of 20 µm.

**Plate 3:**
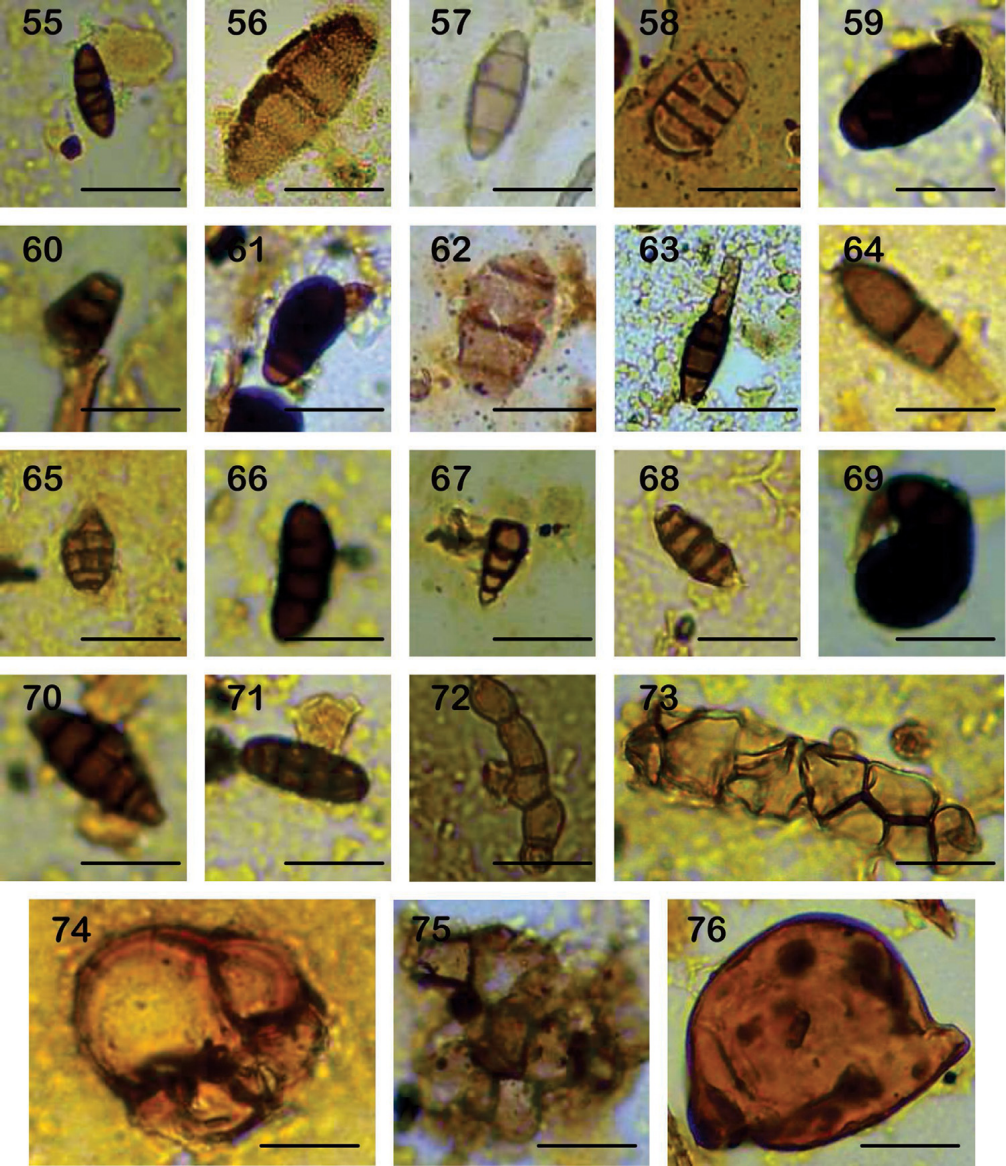
55-65: Ascomycota spores; 66-76: Unidentified non-Pollen Palynomorphs. Scale bar of 20 µm.

This dataset presents a detailed description and environmental affinities of fungal origin NPPs, supplemented with light micrographs to form a descriptive atlas of fungal spores and tissues found in Late Holocene mangrove sediments of the Yucatan peninsula. It is aimed to be used as a reference resource in future paleoenvironmental studies in the Caribbean region. Original counts and Fungal NPPs’ ID from pollen slides can be found in Mendeley's data repository [14]. In the “Morphotypes” sheet, the identified fungal structure is presented. Taxa were named with the abbreviation ECO (El Colegio de la Frontera Sur) followed by a numbering. Individual counts in every sample can be found in “Counts” sheet. Descriptions are organized according to taxa systematics, referring to specific light micrographs in Plates 1, 2, 3.

### 3.1 Fungal spores identification key

**GLOMEROMYCOTA** Division

Class **GLOMEROMYCETES**

Order **GLOMERALES**

Family **GLOMERACEAE**

Genus *Glomus* (Tul. And C. Tul. 1845)

The genus *Glomus* is considered one of the most abundant for having a close symbiosis with the plants of terrestrial ecosystems, they are considered arbuscular vesicle fungi. In paleoecological studies, *Glomus* is used as an indicator of soil erosion processes due to deforestation recorded in lake deposits.

**ECO 2**.- Spore sub globose of 10.2×15.8 µm, verrucose, yellowish, normally solitary, sometimes in pairs, thick-walled up to 1µm (Plate. I, 2).

**ECO 3**.- Spore sub globose of 10.2-11.2 µm, verrucose, yellowish, thick-walled up to 1.1 to 0.8 µm (Plate. I, 3).

**ECO 4**.- Spores of 15.6-6.9 µm, globose, verrucose, yellowish, thin or thick-walled 1 to 0.4 µm (Plate. I, 4).

**ECO 5**.- Spores 14.6-20.1 µm, verrucose, yellowish, usually solitary, thick-walled up 1.1 µm (Plate. I, 5).

**ECO 6**.- Ellipsoid spores 31.6×45.4 µm, subglobose, yellowish brown, verrucose, with subtending hyphae, thick-walled of 2.2 to 1.5 µm (Plate. I, 6).

**ECO 7**.- Spore size of 9.3×16.3 µm, ellipsoid, yellowish, rugose, thick-walled between 16.4×9.3 µm (Plate. I, 7).

**ECO 8**.- Spore of 25.3×28.7 µm, subglobose, verrucose, yellowish, thick-walled between 1.2-0.9 µm (Plate. I, 8).

**ECO 9**.- Spores with globose to cylindrical shapes with verrucose texture. Measurements vary between 28.2-30×27.1-31 µm. They have a yellowish coloration, and the wall has a dark brown color with a thickness of 1 to 1.8 µm (Plate. I, 9).

**ASCOMYCOTA** Division

Class **DOTHIDEOMYCETES**

Order **PLEOSPORALES**

Family **PLEOSPORACEAE**

Genus *Curvularia* (Boedijn 1933)

**ECO 1**.- *Curvularia* sp. With 5 septa of a size of 15.7-17.5×6.8-7.1 µm with a thickness of 0.9 µm and psilate. The presence of *Curvularia* spores in the sediments indicates disturbance by anthropogenic activities and humid environments (Plate. I, 1).

Class **SORDARIOMYCETES**

Order **XYLARIALES**

Family **XYLARIACEAE**

The family Xylariaceae is considered saprophyte or rarely endophytes, in most paleo studies spores have been found in humid environments. Xylariaceae are considered indicators of swamps and soil erosion.

**ECO 10**.- Spore ellipsoid of 6.3×13.4 µm, dark brown, smooth, with rounded tips, with a central germinal line, thin-walled of 0.5-0.8 µm (Plate. I, 10).

**ECO 11**.- Dark brown ellipsoid spore with acute apex, size between 6.6×22.6 µm. It has a defined pore of 0.3×0.4 µm, and thin-walled varies between 0.3 to 0.4 µm (Plate. I, 11).

**ECO 12**.- Cylindrical thin-walled spore with acute apex, a verrucose texture is observed. In addition, dark brown to almost black colors on the shore. The size varies between 13.1×17.8 µm (Plate. I, 12).

**ECO 13**.- Filiform spore with acute apex and a thin germinal line, psilate texture, and light brown color. The size varies between 9×22.7 µm and the wall is 0.5 µm thick (Plate. I, 13).

**ECO 14**.- Ellipsoid Spore of 6.1×10.9 µm, light brown, with rounded apex, smooth, thin-walled (Plate. I, 14).

**ECO 15**.- Fusoid spore with central septum, acute apex, and brown tones. With a psilate texture ranging from 6.6× 12.2, the wall has a thickness of 0.7 µm (Plate. I, 15).

**ECO 16**.- Ellipsoid spore with central septum, dark brown colors rounded apex, and a conspicuous germinal line. Measurements vary between 8.6-10.1×6.7-7 µm, thin-walled of 0.5-0.7 µm (Plate. I, 16).

**ECO 17**.- Ellipsoid spore with acute apexes and central septum, of brown colors, size varying between 7×9.5 µm, presents a dark thin brown to black wall, of 0.5 µm (Plate. I, 17).

**ASCOMYCOTA** Division

**ECO 24**.- Ascomycete conidia, globose in shape with verrucose texture. Normally solitary 7.5 to 16 µm in diameter, dark brown to black (Plate. I, 24).

**ECO 25**.- Conidia globose of 30.7-31.6 µm, dark brown to black, verrucose, with a globose structure up to 9 µm attached to the conidia, dark brown, striated, thin-walled. (Plate. II, 25).

**ECO 26**.- Ascomycete spore of subglobose shape, presents brown colors with darker wall. It has a psilate texture, ranging in size between 18.7-23.5 µm in diameter and thin-walled of 1.3 µm (Plate. II, 26).

**ECO 27**.- Transparent spores with a yellowish edge, belong to the group of ascomycetes. They are found alone or in groups of up to 6 spores. Spores with diameters between 6.5 to 10.3 µm, wall thickness of 0.3 µm. Spores present subtending hyphae of an approximate length of 3.2 µm (Plate. II, 27).

**ECO 28**.- Spore globose of 7-8 µm, dark brown, verrucose, thick-walled. Globular spore of the group of ascomycetes, with a verrucose texture and a yellowish hue, has a thick wall of dark yellow color. The diameter varies between 7 to 8.1µm, thin-walled of 0.9 to 1.9 µm (Plate II, 28).

**ECO 29**.- Globose spore of the group of ascomycetes, from 13.1 to 14.2 µm in diameter, dark brown with a pore of a size of 0.9×1.3 µm. It has a thick wall of 2.1 µm (Plate II, 29).

**ECO 30**.- Globose to oval spore, reddish brown, and verrucose in texture. The diameter varies between 8.3-21.2×6.9-18.1 µm, thin-walled of 0.5 to 0.7 µm (Plate II, 30).

**ECO 31**.- Spore of cylindrical ascomycete with a flattened apex, presents a psilate texture, with subtending hyphae. The size ranges from 20.2-30.6×17.5- 22.3 µm (Plate II, 31).

**ECO 32**.- Cylindrical spore with a flattened apex and verrucose texture. It features dark brown and almost black colors on the wall. The size ranges from 10.3×25.1 µm, with a thickness of 0.8 µm (Plate II, 32).

**ECO 33**.- Globose spore of the group of ascomycetes, with flat apex of brown color of a size that varies between 2×2.6 µm with a thickness of 0.3 µm (Plate II, 33).

**ECO 34**.- Spore globose of 11.1-12.3 µm, dark brown, smooth, with a thick spore wall with two layers (Plate II, 34).

**ECO 35**.- Spores polyhedral of 17.6×19.8 µm, pale brown, with a well-defined pore of 0.3-0.4 µm, thin-walled (Plate II, 35).

**ECO 36**.- Spores ellipsoid of 7.2-9.7×5.8 µm, brown, smooth, septate, thin-walled (Plate II, 36).

**ECO 37**.- Ellipsoid spore of 8.1-16.4×6.1 µm, brown, with rounded tips, smooth, septate, thin-walled of 0.5 µm (Plate II, 37).

**ECO 38**.- Ellipsoid spore of 10.3×22 µm, brown, hyaline at the apex, verrucose, with three septa, thin-walled. The apexes present transparent cells ranging between 2.1-2.3 µm in diameter. The central cells measure between 10.3-13.1× 6.4-7.9 µm and the wall is 0.4 to 0.7 µm thick (Plate II, 38).

**ECO 39**.- Spore ellipsoid of 8.8×9.2 µm, brown, with one central septa, verrucose. Thin-walled of 0.2 to 0.4 µm (Plate II, 39).

**ECO 40**.- Ellipsoid Spore of 12.2×21.1 µm, brown, with rounded tips, with one central septa, rugose, thin-walled (Plate II, 40).

**ECO 41**.- Ellipsoid spores of 14.2×27.3 µm, light brown, psilate, with central septa, thin-walled (Plate II, 41).

**ECO 42**.- Ellipsoid pores of 9.1×11.1 µm, brown, striated, with rounded apex, with central septa, thin-walled (Plate II, 42)

**ECO 43**.- Ellipsoid spores of 18.3-25.8×13.4-15.3 µm, brown, verrucose, with rounded apex, with central septa, thin-walled of 0.6 µm (Plate II, 43).

ECO 44.- Spore ellipsoid of 13.8×15.1 µm, light brown, psilate, with three septa, thin-walled of 0.4 µm (Plate II, 44).

**ECO 45**.- Ellipsoid spore of 12×30.3 µm, light brown, psilate, with rounded apex, with central septa, thin-walled of 0.7 µm (Plate II, 45).

**ECO 46**.- Citriform spore of 18.5×21.6 µm, dark brown, paler at the apex, rugose, with acute and flat apex, with central septa, thin-walled 0.7×1.5 µm (Plate II, 46).

**ECO 47**.- Ellipsoid spore of 10.4×16.8 µm, light brown, verrucose, with rounded apex, with three septa, thin-walled of 0.3-0.4 µm (Plate II, 47).

**ECO 48**.- Ellipsoid spore of 5.3×15.3 µm, dark brown, verrucose, with rounded apex, with three septa, thin-walled of 1.1 µm (Plate II, 48).

**ECO 49**.- Ellipsoid spore of 10.2×11.2 µm, dark brown, smooth, with slightly acute apex, with three septa, thin-walled of 1.5 µm (Plate II, 49).

**ECO 50**.- Conidia ellipsoid of 16.5×35.4 µm, brown, verrucose, with rounded apex, with two septa, thin-walled 0.9 µm (Plate II, 50).

**ECO 51**.- Spore sub cylindrical of 4.3×11.2 µm, light brown, with acute apex, psilate, with three septa, thin-walled of 4.2×11.2 µm (Plate II, 51).

**ECO 52**.- Sub cylindrical spore of 4.2×8.6 µm, light brown, with acute apex, psilate, with three septa, thin-walled 0.2 µm (Plate II, 52).

**ECO 53**.- Spindle spore with 3 septa, belongs to the group of ascomycetes, of yellow tones, of a size of 3.8×13.1, and a thickness of 0.54 µm (Plate II, 53).

**ECO 54**.- Sub cylindrical spore of 4.6×14.1 µm, dark brown, rounded apex, psilate, with four septa, thin-walled of 0.7 µm (Plate II, 54).

**ECO 55**.- Sub cylindrical spore of 4.2×11.8 µm, dark brown, rounded apex, psilate, with four septa, thin-walled of less than 0.5 µm (Plate III, 55).

**ECO 56**.- Ellipsoid spore of 13.9×51.6 µm, light brown rugose, rounded tips, with five septa, thin-walled of 1.2 µm (Plate III, 56).

**ECO 57**.- Ellipsoid Spore of 8.1×16.8 µm, brown, psilate, acute apex, with three septa, thin-walled. Between 0.3 to 0.6 µm (Plate III, 57).

**ECO 58**.- Ellipsoid spore of 13.1×21.8 µm, brown, psilate, rounded apex, with three septa, thin-walled of 0.8 µm (Plate III, 58).

**ECO 59**.- Ellipsoid spore of 8.1×16.8 µm, brown, verrucose, flat apex, with three septa, thick-walled up 1.1 µm (Plate III, 59).

**ECO 60**.- Conidia ellipsoid of 10.7×12.4 µm, brown, psilate, flat apex, with three septa (Plate III, 60).

**ECO 61**.- Ovoid spore of 13.6×24.8 µm, dark brown, rounded apex, with three septa (Plate III, 61).

**ECO 62**.- Ellipsoid spore of 13.2×27.5 µm, light brown, verrucose, rounded apex, with three septa, thin-walled of 0.6 µm (Plate III, 62).

**ECO 63**.- Conidia ellipsoid of 6.1×32.1 µm, brown, verrucose, acute apex, with three septa, thin-walled, with a light brown hyphae at the base. Wall of 0.6 µm (Plate III, 63).

**ECO 64**.- Conidia ellipsoid of 10.2×38. 7 µm, brown, verrucose, with acute apex, with two septa, thin-walled of 0.7 µm with a light brown hyphae at the base (Plate III, 64).

**ECO 65**.- Ellipsoid spore of 10.9×15.8 µm, brown, verrucose, with acute apex, with three septa (Plate III, 65).

### 3.2 Fungal tissues identification key

**ASCOMYCOTA** Division

Class **DOTHIDEOMYCETES**

Family **MICROTHYRIACEAE**

Genus *Microthyrium* (Dems 1841)

The genus *Microthyrium* is considered a saprophytic group, in the few available studies, the presence of these morphotypes indicates conditions of higher humidity.

**ECO 18**.- Dark brown tissue, ostiole is not visible, measuring between 18.5 to 22 µm in diameter, with a wall thickness of 1.1, it has subquadratic cells of a size between 2.3-3.7×2.9-4.3 µm (Plate I, 18).

**ECO 19**.- Dark brown to yellow tissue on the edges, it has subquadratic cells of 2.8×3.5 µm, a wall thickness of 0.8 µm. The approximate diameter varies between 31.1 and 35.3 µm, ostiole is not visible (Plate I, 19).

**ECO 20**.- Ochre brown tissue, with an approximate diameter between 30.7-39.8 µm, the ostiole is not visible, with irregular cells of 2.3×5.4 µm (Plate I, 20).

**ECO 21**.- Tissue 70 µm in diameter, presents a well-defined central ostiole with darker coloration. The cells radiate outward in parallel rows from the ostiole, each subquadratic cell measuring between 2.5×3.2 µm (Plate I, 21).

### 3.3 Hyphae

Hyphae are fungal structures difficult to identify to species level. Authors such as Prager et al. [6] mention that they are preserved in swampy environments or with a high humidity index.

**ECO 22**.- Hyphae 23.8-52.4×3.4-4.2 µm, brown, psilate, thin-walled. (Plate I, 22).

**ECO 23.**- Hyphae 15.7-46.2×2.4-4.2 µm, dark brown, psilate, thin-walled of about 2.4-4.3 µm (Plate I, 23).

### 3.4 Unidentified fungal palynomorphs

**ECO 66**.- Cylindrical spore of 12.1×32.3 µm, dark brown, verrucose, with rounded apex, with three septa, thin-walled (Plate III, 66).

**ECO 67**.- Cylindrical pore of 16. 7 × 4. 9 µm, yellowish brown, psilate, with rounded apex. (Plate III, 67).

**ECO 68**.- Cylindrical spore with flattened apexes, yellowish to dark brown on the wall, has a warty texture. The spore size varies between 8.3×17.8 µm and a wall thickness between 0.9 and 1 µm (Plate III, 68).

**ECO 69**.- Conidia with four septa with shades ranging from light to dark brown, has a psilate texture, conidia have a curvature and are solitary. The size of the conidia varies between 21.2×23.4 µm, the wall thickness is 0.6 µm (Plate III, 69).

**ECO 70**.- Fungal structure of 5 transverse septa, brown and verrucose texture. The size ranges from 12.3×35.6 µm, the wall thickness ranges from 0.5 to 1.2 µm (Plate III, 70).

**ECO 71**.- Spore of 10.8×26.3 µm, brown, psilate, with defined pores of 1.3-1.5 µm, thin-walled is 0.8 µm (Plate III, 71).

**ECO 72**.- Fungal structure of 6.2×31.5 µm, with four septa, yellowish, psilate (Plate III, 72).

**ECO 73**.- Spores globose of 8.9-13.5 µm, brownish, psilate, occurring in clusters of 13-16 spores (Plate III, 73).

**ECO 74**.- Spores globose of 11.6-32.3 µm, yellowish to brownish, verrucose, solitary or occurring in clusters of 5 spores (Plate III, 75).

**ECO 75**.- Globose spore of 8.2-12.5×9.1-13.5 µm, brownish, occurring in clusters of 1 0-13 spores wall thickness is 0.1 to 0.2 µm (Plate III, 74).

**ECO 76**.- Asci 34×53 µm, verrucose, yellowish, solitary (Plate III, 76).

## 4 Experimental Design, Materials and Methods

A 150 cm long sediment core was retrieved in Laguna Cementerio, a coastal lagoon under a subtropical climate regime located in Southeastern Mexico. Mangrove forest surrounds the coring site, and the dominant species are *Rhizophora mangle, Conocarpus erectus,* and *Laguncularia racemosa*. The climate is subhumid, with an average annual rainfall of 1,200 to 1,500 mm, and maximum temperature of 35°C and a minimum of 14°C [15].

The NPP identification and description were performed from sediment samples taken along the core. A total of 56 sediment samples were processed and analyzed. NPPs were extracted using the conventional pollen extraction technique [16,17], using KOH and HCl solutions to remove humic acids and carbonates, respectively. A glycerinated gelatin mounting medium was used for microscope slides.

Identification was performed up to the finest possible taxonomic level using various keys developed for NPPs [[1], [2], [3], [4], [5], [6], [7],[18], [19], [20]]. Morphometric measurements were made as well as the description of each morphotype, the thickness of the wall, color, texture, and shape of the spore or tissue were considered to separate the morphotypes.

## Ethics Statements

This work did not involve any human or animal subjects or experiments

## CRediT author statement

**Cynthia Karina Can-Canales**: Methodology, Investigation, Writing - Original Draft. **Gerald A. Islebe**: Term, Conceptualization, Supervision, Writing - Original Draft. **Alicia Carrillo-Bastos**: Supervision. **Nuria Torrescano-Vale**: Supervision, Resources. **Alejandro Antonio Aragón Moreno**: Data curator, Visualization, Writing-Review & Editing.

## Data Availability

Non-Pollen palynomorphs: Fungal spores and tissue of Caribbean mangroves, Southeastern Mexico (Original data) (Mendeley Data)
